# Supporting Novice Nurses’ Transition to Independent Practice: Evaluation of the TIPS Program Using the Kirkpatrick Model

**DOI:** 10.3390/nursrep15020050

**Published:** 2025-02-01

**Authors:** Charissa Cordon, Desa Dlugosz, Lorena Lopez, Rona Gelacio, Kate Smith-Eivemark, Shannon Maier, Amir Ginzburg, Kevin Hua, Dian Williams, Terri Irwin

**Affiliations:** 1 William Osler Health System, Brampton, ON L6R 3J7, Canada; charissa.cordon@williamoslerhs.ca; 2 Lawrence Bloomberg Faculty of Nursing, University of Toronto, Toronto, ON M5T 1P8, Canada; 3 School of Nursing, McMaster University, Hamilton, ON L8S 4K1, Canada; 4 Trillium Health Partners, Mississauga, ON L5B 1B8, Canadarona.gelacio@thp.ca (R.G.); kate.smith-eivemark@thp.ca (K.S.-E.); kevin.hua@thp.ca (K.H.);; 5 Temerty Faculty of Medicine, University of Toronto, Toronto, ON M5S 1A8, Canada

**Keywords:** nursing administration, team-based care, unregulated staff, mixed-care models, staffing mix, extenders

## Abstract

Trillium Health Partners (THP) is a hospital network that serves the Mississauga region of Ontario, Canada, and sees nearly 1.7 million patient visits each year. THP is also a provider of highly specialized services to the region and a fully-fledged academic teaching center, with embedded research and innovation. **Background/Objectives**: Highly trained, skilled, and experienced nurses are foundational to THPs ability to meet the complex care needs of our patients across specialized programs. In 2024, 50% of the nursing workforce at THP was noted to have less than five years of experience. This generation of nurses are reporting high levels of burnout and are at greater risk of leaving the profession. The more experienced nurses are also facing burnout due to the continued pressures and demands in the workplace, having to manage an increasingly complex patient assignment, while providing mentorship to more novice nurses. Based on the existing literature and our collective experience at THP, we have developed the Transition to Independent Practice Support (TIPS) program, a multi-pronged approach to bolster support for our nursing workforce. The TIPS program at THP is designed to address knowledge gaps and enhance clinical competence among new and aspiring nurses. The primary aim of this study is to evaluate the efficacy of the TIPS program in facilitating the transition of novice nurses to independent and competent practitioners, utilizing the Kirkpatrick Model of Evaluation The specific objectives include (a) assessing participants’ reactions to the TIPS program; (b) measuring the increase in nurses’ knowledge and confidence following program participation; (c) evaluating changes in nurses’ behavior and practice post-program; and (d) determining the program’s impact on nurse retention and quality of patient care. **Methods**: Grounded in Meleis’s Transition Theory, the program combines webinars, simulations, clinical rounding, and mentorship to build resilience, decision-making, and confidence. Evaluative measures, based on the Kirkpatrick Model, assessed participant satisfaction, learning outcomes, behavior changes, and retention. Results: From September 2023 to March 2024, 388 interdisciplinary participants attended the program, including nursing students (56%), registered nurses (24%), registered practical nurses (14%), and aspiring nurses in other interprofessional roles (6%). Participants expressed high satisfaction with the program, achieving a mean reaction score of 4.80 (SD = 0.2921). Ninety-five percent found the sessions relevant, and 98% rated the facilitators as knowledgeable. Self-reported confidence significantly increased across all topics, with overall mean confidence scores rising from 2.94 to 4.52 (*p* < 0.0001, Cohen’s d = 3.01), demonstrating a strong impact on skill acquisition and application. Behavioral evaluations through simulations, competency checklists, and follow-up touchpoints confirmed improved clinical performance, with participants achieving 100% compliance to nursing skills during simulations and sustained confidence at three-month follow-ups. Since the program’s implementation, nursing turnover rates decreased from 9.52% to 7.7% by March 2024, with 100% of RNs, RPNs and IENs of TIPS participants retained within six months. **Conclusions**: This paper outlines the TIPS program and the diverse teaching and learning methodologies used in the design and program implementation to ultimately support the transition experience of the new gradate nurse into acute care. Preliminary outcomes are discussed including nursing retention rates, nurses’ knowledge, confidence, and skill levels.

## 1. Introduction

Trillium Health Partners (THP) is the hospital network that serves the Mississauga region of Ontario, Canada and sees nearly 1.7 million patient visits each year. THP is also the provider of highly specialized services in the region, and a fully-fledged academic teaching center, with embedded research and innovation [1,2].

Highly trained, skilled, and experienced nurses are foundational to THP’s ability to meet the complex care needs of our patients across specialized programs. 50% of the nursing workforce at THP have less than five years of experience and are under the age of 29 [1]. Opportunities exist to further strengthen and develop new nursing programs aimed at stabilizing and supporting our novice nursing workforce to ensure safe and independent transition to practice. Investing in our novice workforce ensures that we retain skilled nurses at THP, especially in highly specialized areas, such as critical care and emergency medicine.

Using the Kirkpatrick Model for evaluating training effectiveness [3], the TIPS program has demonstrated remarkable success in supporting THP’s new nurses, and it has enhanced their communication, delegation skills, critical thinking abilities, and overall competence, facilitating a smoother transition and empowering them to practice to their full scope.

## 2. Background

The Registered Nurses Association of Ontario (RNAO) recently reported on the impact of the COVID-19 pandemic on the nursing workforce. Their report emphasized that early career registered nurses (RNs) reported the greatest challenges with coping and transitioning into their role as regulated providers. The report highlighted Ontario’s risk of losing more than 20% of early career RNs. In fact, 69% of survey respondents reported planning to leave their current position within the next five years [4]. Similarly, the Registered Practical Nurses Association of Ontario (WeRPN) reported nearly one in two RPNs (47%) are considering leaving the profession [5]. In one study that explored reasons why new graduate nurses are leaving the profession in their first year of practice, the most common causes included interpersonal violence, workplace stress, feelings of incompetence, low confidence related to practice, unit workload, and interdisciplinary relationship [6,7].

THP employs over 4600 nurses, most of which have less than five years of experience [1,2]. During the pandemic and the nursing crisis, THP was challenged with a high vacancy rate. Despite innovative recruitment efforts and significant pandemic-associated impacts to human health resources, nursing vacancies increased from an average of 5.6% (2017–2020, pre-pandemic) to 11.1% (2021–2023, pandemic). Although the nursing vacancy rate improved during pandemic recovery to 7.7%, THP continued to be challenged with a relatively inexperienced workforce. Newly graduated nurses rely on the mentorship from more experienced nurses; however, experienced nurses are finding it challenging to provide mentorship while also caring for an increasingly complex patient case mix. The need to provide support to novice nurses and help them transition safely and effectively into independent practice was THP’s top priority. This led to the development of the Transition to Independent Practice Support (TIPS) program, offering mentorship and clinical guidance to THP’s new and aspiring nurses, facilitating their safe and seamless transition from academia to independent clinical practice.

The primary aim of this study is to evaluate the efficacy of the Transition to Independent Practice Support (TIPS) program in facilitating the transition of novice nurses to independent practice, utilizing the Kirkpatrick Model of evaluation. The specific objectives include (a) assessing participants’ reactions to the TIPS program; (b) measuring increase in nurses’ knowledge and confidence following program participation; (c) evaluating changes in nurses’ behavior and practice post-program; and (d) determining the program’s impact on nurse retention and quality of patient care.

## 3. Review of the Literature

New nurses’ feelings of optimism and excitement can quickly turn into an overwhelming sense of exhaustion and doubt as they navigate a fast-paced care environment with high patient acuity and staffing challenges, all while trying to apply theoretical knowledge and technical skills to the practice environment. In fact, in a longitudinal study conducted with new graduate nurses, prior to starting practice without supervision (independent practice), new graduate nurse participants reported high professional confidence and competence in their ability to apply theory to practice and work under pressure. However, after 1 month of independent practice, respondents reported a decrease in this self-perception, experiencing feelings of uncertainty with their practice [8]. Technical skills have frequently been considered a set of simple task-oriented competencies; however, the performance of these skills is a complex action requiring knowledge, skill, judgement, and the anticipation of both expected and unexpected outcomes [9]. Meleis’s Transition Theory proposes that this complex and challenging professional transition can lead to heightened exposure to stress and anxiety, which may compromise the new graduate’s physical and psychological health [10]. This may also lead the new professional into a state of Transition Shock, which is when an individual experiences feelings of anxiety and knowledge insufficiency as they enter a new role or environment; Transition Shock is associated with poor job satisfaction and low retention rates [11,12,13].

Meleis’s Transition Theory emphasizes the importance of evaluating the transition experience, including change triggers, properties, conditions, and patterns of response, as well as the interventions to support a seamless transition [10]. There are several factors that impact the nuanced transition of new healthcare professionals, including the ability to adapt to specific workplace systems, structures, and expectations; self-perception of confidence and competence for the new role; attitudes and competencies developed based on practice exposure and experience; and ability to manage challenges as they arise [8,14]. Practice transition is further affected by the availability of clinical support and ability to receive timely feedback; professional development opportunities focusing on mentorship, clinical competence, and cultural diversity; and complexity of the work environment [8,13].

Developing support at points of transition that account for these factors is therefore crucial for the facilitation of safe and effective practice and preservation of nurses’ wellbeing. Although no ideal curriculum exists to support this transition, key recommendations in the literature include the development of orientation programs that span over the first year of hire, frequent and structured check-ins with their local leadership, availability of structured education residency programs aimed at developing clinical confidence, and availability of mentors [7,13,15,16,17].

**Figure 1:**
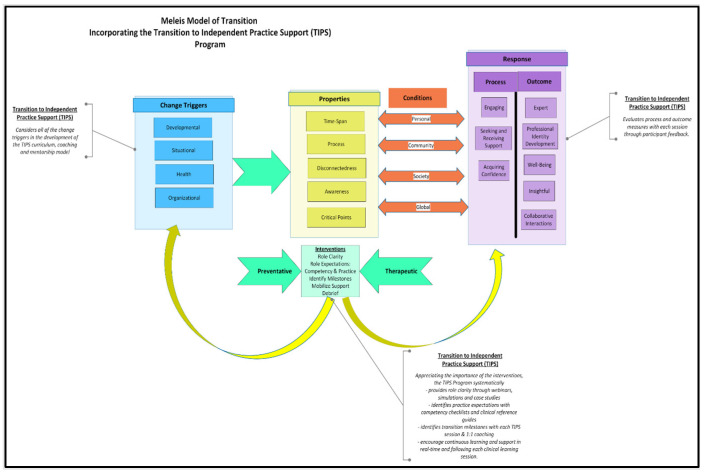
Meleis Model of Transition adapted for the Transition to Independent Practice Support Program [10].

Much of THP’s current nursing workforce comprises newly graduated nurses, internationally educated nurses (IENs), and nurses with previous experience who are developing new skills required for their clinical practice environment. Each of these nursing groups responds differently to change triggers and the conditions and properties impacting them. The TIPS program was designed to support these nurses through points of transition by leveraging the Meleis’s Transition Theory. Meleis’s Transition Theory emphasizes that irrespective of the type of transition experienced, all transitions should be understood as a continuum as opposed to a singular event and, likewise, should be approached with long-term, evolving solutions that adapt to learners’ needs [10,18]. More specifically, it includes support that allows for gradual adjustment to their new environment, such as continuous learning opportunities allowing for real-time feedback, setting practice expectations, providing role clarity, and mentorship opportunities [10,18]. As demonstrated in Figure 1, TIPS employed several of these strategies through clinical rounding with novice staff where, in the moment, support and feedback was adjusted to the needs of the new hire and situation [10]. Another key strategy utilized, as suggested by Meleis, was the ability to attend educational events over the course of their first year of hire versus only a single event, supporting an environment of continuous learning and allowing for gradual adaptation [18]. To adjust to the needs of the target audience at THP, additional findings from the literature were also used in the development TIPS, including (1) where and how to navigate workplace processes and structures to support their transition at THP specifically, (2) the development of self-perceived confidence to ensure they feel capable and competent in the delivery of safe care, and (3) fostering of resiliency strategies to navigate difficult and rapidly changing environments [10,14].

## 4. The Transition to Independent Practice Support Program

### 4.1. Evolution of the Program: The TIPS Facilitator Role

Despite pre-existing resources at THP, a need to centralize education was identified to ensure a more equitable offering of services and standardization of content across departments. At the same time, impacts from the pandemic placed requests for greater clinical mentorship and support beyond standard business hours by several frontline nurses, clinical managers and educators.

To address this complex need, in January 2021, the department of Professional Practice introduced the role of the Professional Practice Clinical Educator (PPCE), whom along with leading corporate initiatives like orientation, would be responsible for clinical rounding and provide at-the-elbow support through an on-call service to point of care staff during the hours of 1600–2300 on weekdays, and 0800 to 2300 on weekends. This team originally consisted of four registered nurses (RNs) who were selected based on experience with formal clinical teaching, preceptorship, and mentorship, as well as post-graduate certifications in adult learning principles and simulation science. As the role evolved to include larger corporate program initiatives, such as onboarding of clinical staff, and supporting IENs’ transition to Ontario, the PPCE team grew to a group of 10 RNs, ensuring support and educator coverage across THP. Acknowledging that new nurses need structured programs to enhance confidence and promote development in their transition to independent practice, in September 2023, THP launched an evidence-informed initiative to support all new and aspiring nurses at THP: the TIPS program.

**Figure 2:**
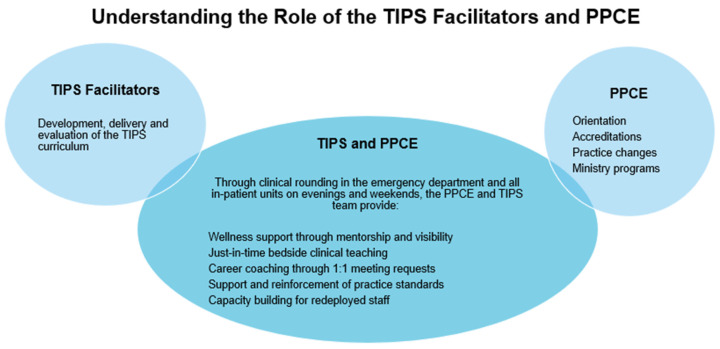
Understanding the role of the TIPS facilitators and PPCE.

To best meet the varying needs of the participants and program demands, two of the PPCEs took on the role of leading the Transition to Independent Practice Support (TIPS) as facilitators, responsible for program development, delivery, and evaluation. In addition to this, alongside the PPCE, the TIPS facilitators continued to provide at-the-elbow support to clinical staff on evenings and weekends through clinical rounding and an on-call service (refer to Figure 2).

### 4.2. Evolution of the Program: The Curriculum

The original curriculum was developed using learning patterns and gaps identified through clinical rounding and from anecdotal reports from frontline staff and leadership. This led to the development of content that primarily reviewed anatomy and physiology, interventions, nursing management, interprofessional collaboration and documentation practices. Although the first cycle of the educational curriculum (2023–2024) was successful with 388 participants and several positive reviews from end-users and stakeholders alike, a formative evaluation of the program gave insight into the more specific learning needs of the new and aspiring nurses at THP. Formative evaluative efforts included open-ended surveys; interviews with participants, preceptors, and local leadership; and purposeful interactions with random participants two months after the first cycle program. Data demonstrated that most attendees had a strong introductory understanding of anatomy, physiology, and nursing management; however, the critical application of these concepts was lacking in the presence of abnormal findings. The data evaluation was further supported by firsthand experience from clinical rounding and data collection of calls received from unit staff. The data revealed that this group of nurses required increased coaching regarding decision-making, clinical judgement, and reasoning in the presence of abnormal findings and patient deterioration. Alongside these findings, anecdotal insights from facilitating sessions informed the second cycle of the TIPS educational curriculum (2024–2025).

The second cycle of TIPS was adapted to more strongly support the development of critical thinking and reasoning. This led to a change in the learning objectives, with greater emphasis on physical examination techniques, nursing diagnostic reasoning, prioritization in the presence of abnormal findings, and interprofessional collaboration and communication. The formative feedback also informed the changes made to the delivery of sessions, with frontline requesting that more sessions be hosted in the evenings and weekends and that greater emphasis be placed on simulation events. Summative feedback was and continues to be completed based on the principles of the Kirkpatrick Model (2016), which is discussed in greater detail in succeeding sections of this paper [19].

### 4.3. The TIPS Program: Current State

In its most current state, the TIPS program continues to be led by two TIPS facilitators, who are responsible for the following:

1. Development of curriculum, educational content (monthly webinars and simulations), and clinical reference guides, summarizing key learnings for the month.
2. The creation of competency checklists, which guide the facilitators’ assessment of the learner’s performance and are shared with the learner for personalized professional development.
3. Maintenance and upkeep of a centralized education page on the hospital intranet, where all corporate educational events at THP are posted, as well as a specific TIPS webpage, where all TIPS content are made available to all THP staff
4. Organization of 1:1 meetings with program participants for the purpose of career coaching and mentorship
5. Providing at-the-elbow support to clinical staff through rounding on evenings and weekends (shared task with PPCE, refer to Figure 1)
6. Formative and summative evaluation of program

### 4.4. The TIPS Program: Educational Content

TIPS program delivers a curriculum comprising monthly webinars and simulations, utilizing both high- and low-fidelity methods. For every webinar hosted, two to three simulation events are developed for the learner to apply, integrate, and deepen their understanding of the material. The webinars function as preparatory material, giving learners the essential clinical knowledge required for the simulations. To best adapt to nurses’ varying clinical schedules, all webinars were recorded and posted on a centralized intranet page, in addition to all learning materials.

The sequence for the simulations typically involved the learner receiving a report, conducting a comprehensive head-to-toe assessment, encountering a non-critical patient deterioration necessitating an escalation of care, experiencing a subsequent critical deterioration requiring more intensive intervention, and culminating in a code blue scenario. All simulations were facilitated in consultation with an educator with formalized training in simulation delivery. Upon completion of each case, a debriefing session was conducted using the Promoting Excellence and Reflective Learning in Simulation (PEARLS) framework [20]. This framework incorporates (1) learner self-assessment, (2) targeted facilitation to foster critical reflection, and (3) directive performance feedback [20]. The PEARLS framework facilitated a structured debrief aimed at enhancing both technical skills and teamwork. To facilitate professional development, a completed competency checklist that detailed session objectives and learner progress was provided to all participants. Additionally, a clinical reference guide was provided to participants following each simulation summarizing key learnings that month.

### 4.5. The TIPS Program: Clinical Rounding

This segment of the program arm provides “at the elbow” or “at the bedside” support for clinical staff, with an emphasis on novice and aspiring nurses. In tandem with the PPCE, the TIPS facilitators provide clinical rounding on all in-patient adult medicine units and specialty programs, including cardiovascular, surgery, rehabilitation, geriatrics, mental health, pediatrics, women’s health, emergency medicine, and critical care, during evenings and weekends. Currently, there are seven PPCEs with rotating day and evening schedules, providing after-hours and weekend support, and they can be accessed by direct pages through THP’s switchboard. In 2023, the PPCEs received a total of 3122 pages (~260 per month), with 1383 pages (115 per month, approximately 44% of the total) occurring on weekends.

The TIPS program’s off-hours coverage addressed nine major themes through its rounding efforts: Mentoring and Career Navigation (26 instances—services designed to aid individuals in exploring their interests, identifying career goals, and formulating action plans); Redeployment of Staff (42 instances—providing in-person support to nurses reassigned to unfamiliar units during staff shortages, aiding their adjustment to new environments and patients); Organizational Initiatives (55 instances—addressing accreditation standards and other initiatives implemented by THP); Support for IEN, Externs, and Novice Learners (98 instances—facilitating orientation, learning, and professional development for specific staff members); Emotional Support (125 instances—activities aimed at fostering a positive work environment conducive to wellness and psychological safety); Locating Supplies (140 instances—assisting units in finding and delivering supplies and medications); Resource Navigation (195 instances—helping staff locate and utilize relevant resources, and promoting adherence to policies and procedures); Just-In-Time Teaching (959 instances—supporting frontline staff with new or unfamiliar clinical skills while emphasizing best practice guidelines); and Support for Staff (1324 instances—contributing to the learning and development of staff, supporting bedside care, high-acuity situations, and ensuring the delivery of safe, high-quality patient care) (see Appendix A for rounding themes and page totals).

Through clinical rounding, the TIPS facilitators also connect with clinical staff to arrange 1:1 meetings for mentorship and career coaching purposes, as requests arise. Although preference is given to new and aspiring nurses, this support is open to all clinical staff. Along with the PPCE team, TIPS program is designed to address the support deficit that arises in the absence of unit educators, particularly given the novice nursing workforce and the extended knowledge gaps that extend beyond the standard Monday to Friday operational hours of unit educators.

## 5. Materials and Methods

### 5.1. Participants

Inclusion criteria for the TIPS program include registered nurses (RNs), registered practical nurses (RPNs), and internationally educated nurses (IENs) who are newly licensed in Ontario, Canada, and/or within a specific period (e.g., 1–2 years post-graduation). In addition, aspiring nurses, such as nursing students and/or clinical externs in any year of study in a College of Nurses of Ontario (CNO)-approved Baccalaureate Nursing (BScN or BN) program and Practical Nursing (PN) program, were included.

Exclusion criteria were non-nursing professionals, or nurses with >5 years clinical experience, unless they are transitioning to another specialty area.

The TIPS program webinars and simulations were voluntary for participants to attend. Hence, attendance to sessions was often during their break or personal time. As such, a voluntary sampling method was used and relied on participants expressing interest in joining the program. However, several methods were used to promote the TIPS program and invite nursing staff and students to attend. Communication was reiterated via e-mail to all clinical educators to promote the program to their staff. The TIPS facilitators also created a poster that promoted each event, with dates to the events and QR codes for registration; posters were displayed on every unit as an additional layer of program promotion (refer to Appendix D for TIPS Poster Phase 1 and Appendix E for TIPS Poster Phase 2). Lastly, communication via e-mail was sent out to clinical instructors and preceptors who are scheduled to have a group of nursing students on medical units during fall or winter terms. In addition to using the online platform, PPCEs also promoted the program during clinical rounding on evenings and weekends, talking with nursing staff face-to-face.

### 5.2. Measurements

Quantitative data collection included a demographic survey and pre/post-questionnaires (Appendix D) designed to evaluate the educational impact of the TIPS program. These questionnaires assessed learners’ self-perceived effectiveness of the program content, facilitator style, and applicability to clinical practice. The TIPS questionnaires were adapted from Heydari (2019), who utilized pre- and post-assessment surveys grounded in the Kirkpatrick model domains to evaluate the effectiveness of educational initiatives for healthcare workers [21].

In Heydari’s (2019) study, the surveys demonstrated strong psychometric properties, with a reported reliability coefficient (r = 0.83), indicating they were valid and robust instruments [21]. Minor adaptations were made to Heydari’s surveys to tailor them to the audience at THP, ensuring relevance while maintaining the integrity of the original tool [21].

To analyze the data, descriptive statistics (percentages, means, and standard deviations) were used to summarize participant responses. Paired *t*-tests were conducted to evaluate pre/post-changes, and effect sizes were calculated using Cohen’s d to assess the magnitude of these changes. These statistical methods provided a comprehensive understanding of the program’s impact.

### 5.3. Evaluation Framework: The Kirkpatrick Model

The Kirkpatrick Model (2016), which was used in this study, is one of the most widely used frameworks for evaluating the effectiveness of training and development programs [19]. It is a validated tool because it has been extensively researched and refined over time to provide a structured approach for measuring the impact of training initiatives. Originally developed by Donald Kirkpatrick in 1959, the model is considered effective in assessing multiple dimensions of program success, from participant satisfaction to real-world outcomes [19]. It consists of four levels of evaluation, each designed to assess different aspects of a training program’s impact: (1) reaction, evaluating the degree to which individuals find the program engaging and relevant to their role; (2) learning, which measures the degree to which the individuals gain the intended knowledge, skills, attitude, confidence, and commitment resulting from their participation; (3) behavior, which measures application of their learning to their clinical practice; and (4) results, evaluating targeted outcomes that occur as a direct result of the education [19,22]. The TIPS program has integrated all levels of the Kirkpatrick Model into its evaluation process through the utilization of questionnaires and checklists, strategically given to the participants at different points of the program, further described in succeeding sections on data collection [19,22].

### 5.4. Data Collection

#### 5.4.1. Reaction (Level 1)

Level 1 measures participants’ initial reactions to the program (e.g., satisfaction, engagement, and relevance). Participants completed a satisfaction survey immediately after each webinar and simulation, with questions like “How satisfied were you with the program?” and “Was the information relevant to your role?” An online survey utilizing a 5-point Likert-type scale from 1 (strongly disagree) to 5 (strongly agree) was used (refer to Appendix F for full list of survey questions used). The responses provided quantitative data on overall satisfaction, while open-ended questions added qualitative insights on how the program can be improved and its relevance to their practice.

#### 5.4.2. Learning (Level 2)

Level 2 measures what participants have learned during the training, i.e., the increase in knowledge, skills, and confidence because of the program [20]. For our program, we specifically focused on self-reported confidence levels of participants with regard to nursing skills outlined within the TIPS program (refer to Appendix B and Appendix C for all topics covered). We chose to focus on confidence because research suggests that it directly influences job satisfaction, long-term retention, and engagement. Structured programs such as TIPS can significantly increase self-reported confidence, leading to improved retention rates and satisfaction [23,24]. A survey (Appendix F) using a five-point Likert-type scale, from 1 (poor) to 5 (excellent), was used to assess their confidence in their ability to perform the nursing task and ability to seek out support or further education in their independent practice. The learners completed the surveys before the start of the session and immediately after the session’s completion. In addition to pre- and post-surveys, the facilitators included knowledge test questions throughout the webinars to facilitate discussions with the learners. Statistical analysis (paired *t*-tests) was conducted to compare pre- and post-test results, validating learning outcomes and confidence in skills.

To further quantify the effect size of the intervention, Cohen’s d was calculated. Cohen’s d measures the standardized difference between two means, allowing us to assess the magnitude of the change in confidence levels pre- and post-training. An effect size of 0.2 is considered small, 0.5 medium, and 0.8 or higher large [25]. In this study, the Cohen’s d values reflected a large effect size, highlighting the significant impact of the TIPS program on participants’ self-reported confidence levels.

#### 5.4.3. Behavior (Level 3)

Level 3 examines how well participants apply their learning in the workplace. After 2–4 weeks of completing a specific topic webinar, participants’ behavior was evaluated in a simulated clinical setting, using an observation competency checklist. TIPS facilitators use a competency check list to assess how often participants demonstrate the learned behaviors in the simulated clinical setting and provide structured feedback on critical thinking and skill application. Participants also demonstrated the teach-back method, and in-the-moment remediation was provided as needed. In addition, participants were given opportunities to practice new skills, ask questions, and clarify any expectations or relevance to practice [19].

#### 5.4.4. Results (Level 4)

Evaluation of program results focused on the retention of nursing staff. Measurement included monitoring staff turnover rates, with the aim of decreasing recruitment demand. Targeted outcomes include the conversion of at least 75% clinical externs (employed nursing students completing an externship at THP), newly graduated nurses, IENs, and nurses with less than three years’ experience working in specialized areas.

### 5.5. Ethical Consideration

This quality improvement (QI) project was conducted in full adherence to the relevant local legislation and guidelines that define and regulate QI activities in Ontario. According to the Ontario Health guidelines, quality improvement projects fall outside the scope of research and do not generally require formal review by a Research Ethics Board (REB), as they are designed to assess and improve healthcare services within the context of an organization. Specifically, under Article 2.5 of the “Tri-Council Policy Statement: Ethical Conduct for Research Involving Humans—TCPS 2 (2022)—Chapter 2: Scope and Approach” [26], QI activities, such as performance assessments and program evaluations, are not considered research, provided they are used exclusively for internal improvement and not for generalizable knowledge.

However, recognizing the importance of ethical oversight, this QI project was retrospectively submitted to Trillium Health Partners’ Research Ethics Board (REB#263) for review. This was performed to ensure full transparency and to confirm that the project adhered to ethical standards, including ensuring the protection of participant confidentiality, voluntary participation, and the appropriate use of collected data. The REB review was sought in alignment with best practices in quality improvement to demonstrate a commitment to upholding ethical principles, even when formal review may not be required by policy.

Participants in this QI project were informed that their involvement was voluntary, and they had the option to withdraw at any time, without consequence. As the project did not involve research as defined by REB policies, explicit informed consent was not required; however, consent was implied through voluntary participation. Data were anonymized and presented in aggregate form to protect participant privacy, and the results were used solely for quality improvement purposes, in compliance with local privacy and data protection laws.

The retrospective approval from the REB affirms that this project was conducted as a quality improvement initiative, with appropriate ethical considerations in place, ensuring that it met the necessary standards for both ethical practice and regulatory compliance.

## 6. Results: Using the Kirkpatrick Model

In Phase I of the TIPS program, 388 participants attended the webinars and simulations. Data results were collected from September 2023 to March 2024.

The participant sample consisted of primarily nursing, of which more than half (56%) were nursing students, 24% were registered nurses (RNs), and 14% were registered practical nurses (RPNs). However, a small percentage of participants (6%), consisted of aspiring nurses currently in other interprofessional roles, such as unit clerks, personal support workers (PSWs), and clinical instructors. Almost half (46%) of participants were noted to be <30 years old, 32% were between the ages of 30 and 40 years old, 14% were between the ages of 40 and 50 years old, and 8% were 50 years old and above. Over a third (35%) of program participants had less than 1 year of nursing or work experience, 17% had 1–3 years of experience, 12% had 3–5 years, 19% had 5–10 years, and 17% had more than 10 years of work experience.

Given that the program is still in its preliminary stages, it is important to highlight that only 171 participants completed all four levels of evaluation and are confirmed to still be employed within THP. Due to resource constraints, the retention rates for all nursing students who participated in the program are not currently available.

### 6.1. First Level of Evaluation: Reaction

Descriptive statistics show a mean reaction score of 4.80 (with 5.0 being the highest possible score), indicating high satisfaction among the participants with the training program. The standard deviation (SD) of 0.2921 indicates that the ratings for each session are relatively consistent across participants. This suggests low variability in how participants felt about the training.

**Figure 3:**
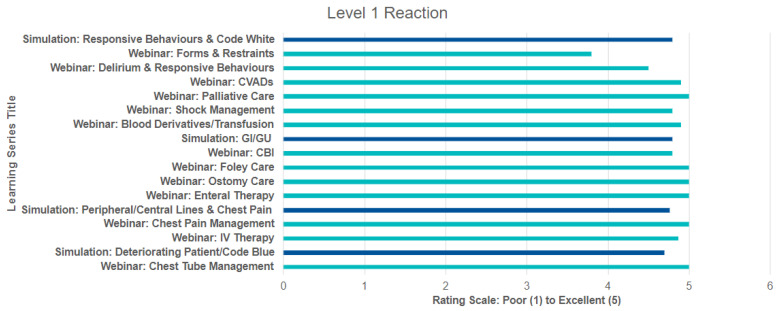
Level 1 reaction graph.

The first level of evaluation measured how much the study participants reacted favorably to the TIPS program (webinar and simulation). As noted in Figure 3, the reaction from participants after each education session was positive: participant satisfaction was rated at 90%, 94% found the educational content was well organized and informative, 95% found the sessions relevant to their jobs, and 98% thought that the facilitators were knowledgeable and presented the content clearly.

The consistency of the scores (indicated by the low SD) and the high mean reaction score (4.80) provide evidence that the program was well-received overall.

The forms and restraints session, with a mean of 3.8, shows that there is some variation in participant reactions, and this topic might benefit from further refinement. However, with a small SD across the rest of the sessions, the overall program appears to be successful.

### 6.2. Second Level of Evaluation: Confidence

As noted in Table 1, self-reported confidence in skills increased from pre-evaluation (M = 2.9, SD = 0.5) to post-evaluation (M = 4.5, SD = 0.55; two-tailed *p*-value less than 0.0001). The confidence intervals (CIs) for the mean differences (e.g., [1.51, 1.65] for the total program) indicate that the observed improvements in scores are statistically significant and not likely due to chance. The effect sizes (Cohen’s d) for individual topics and the overall program suggest large to very large effects, with values such as 1.90 for deteriorating patient/code blue SIM (for example) and 3.01 for the overall program, indicating that the training had a strong impact on participants’ self-reported confidence levels.

The TIPS Phase 1 program has demonstrated a significant improvement in knowledge and skills among nurses, aligning with Level 2 of the Kirkpatrick Model. The post-test results, backed by statistical significance (confidence intervals and effect sizes), suggest that the nurses not only learned new information but also became more confident in applying it in practice. The program’s impact is substantial, indicating that it successfully achieved its learning objectives and contributed to the competence of new nurses at Trillium Health Partners.

### 6.3. Third Level of Evaluation: Behavior

Behavior change was assessed through a combination of simulation performance, competency validation by TIPS facilitators, and follow-up check-ins at 3 months post-program.

*Simulation performance:* Two weeks after completion of the webinars, participants engaged in simulations designed to reflect real-world clinical scenarios. Confidence levels, as self-reported by participants prior to the simulation, showed a marked increase compared to pre-webinar baseline measures. The simulations focused on specific nursing skills emphasized in the TIPS program. Notably, participants achieved a 100% compliance score during these simulations, indicating full adherence to the prescribed protocols and best practices.

*Competency validation:* Following the simulations, participants were provided with competency checklists completed by TIPS facilitators. These checklists evaluated the participant’s competency in performing key nursing skills. The checklists were designed to assess both technical and behavioral aspects of nursing practice, such as communication with patients and colleagues, adherence to safety protocols, and clinical decision-making. Data from these competency assessments were used to validate the real-world application of skills acquired through the TIPS program. 

### 6.4. Fourth Level of Evaluation: Results

The TIPS program began in September 2023, and nursing turnover rates decreased beginning in December of 2023. In the context of an organization-wide turnover rate of 9.52%, 100% of RNs, RPNs, and IEN participants remained in the organization within 6 months of completion of the program. Since the deployment of the TIPS program, along with other retention strategies, the hospital’s turnover rate also decreased to 7.7% in March 2024. The results demonstrate the efficacy of the TIPS program, as well as its success in reaching target outcomes of reducing nurse turnover and consequently, increasing nurse retention.

### 6.5. Qualitative Data

The feedback from participants regarding both the webinar and the accompanying simulation was highly positive, with several key themes emerging from the qualitative data.

#### 6.5.1. Webinar Reaction

Immediately following each webinar, participants were asked, “Is there anything that you would like to be done differently in future webinars?” Participants expressed strong satisfaction with the webinar’s format, appreciating the clarity and effectiveness of the content delivery. A majority of respondents emphasized the concise and structured nature of the presentation, with one participant noting, “I love how the webinar is only an hour; the information was short and easy to understand”. Several participants highlighted the informative nature of the session, as one remarked, “The webinar was great and very informative!” However, there was a recurring request for more interactive elements, with participants suggesting, “More interactive discussions and questions please”, and “I would like practice real-life scenarios to improve my critical thinking”. These comments indicate that, while the content was well received, participants could benefit from a more engaging and participatory learning experience.

Additional suggestions for content improvement included requests for more case studies, videos, and visual aids. One participant stated, “Another case study/scenario-outlining sequential steps”, while others emphasized the need for videos or pictorial demonstrations, with one participant noting, “More videos”. Some participants also requested a slower pace for critical topics to ensure clarity, with one suggesting, “Go a little slower over important details”. Moreover, there were calls to expand the content to other healthcare team members, reflecting a broader interest in disseminating the information across the organization: “Should be offered to other members of the health team”.

#### 6.5.2. Post-Simulation Reaction

At the end of each simulation, participants were asked, “What did you enjoy the most about the webinar and accompanying simulation?” Participants overwhelmingly appreciated the hands-on, realistic nature of the simulation. The interactive, practical approach allowed participants to engage deeply with the material, especially in emergency scenarios, like code blue and CPR. One participant highlighted, “I enjoyed how engaging and hands-on this simulation was”, while another shared, “The hands-on practice on the manikin”. The simulation was widely praised for its realism, with participants commenting, “I love how realistic the simulation is in terms of a deteriorating patient”, and “It was well arranged and felt like a real case scenario”. These comments demonstrate that the simulation successfully replicated real-life situations, enhancing the learning experience.

The safe, supportive, and nonjudgmental environment fostered by the facilitators was another key strength of the session. One participant noted, “Loved how you created a safe and non-judgmental environment”, while another emphasized, “The team that was conducting the session was super supportive!” The judgment-free atmosphere allowed participants to practice their skills with confidence, contributing to a positive and productive learning experience.

#### 6.5.3. Teamwork and Skill Development

Teamwork and collaboration were recurring themes in participants’ feedback. The group setting allowed for coordinated efforts and knowledge sharing, with one participant commenting, “Working as a group/felt more confident after attending the simulation”. Another noted, “Great teamwork, explanation before the sim”. These insights suggest that the simulation fostered a sense of collective learning, where participants could rely on each other and work together to solve problems. 

Many participants also emphasized the educational value of the simulation in enhancing their skills. The session was particularly beneficial for learning critical emergency procedures, such as code blue management and CPR. One participant shared, “Expanding my knowledge of code blues and practicing skills in sim”, while another remarked, “Learned new skills and wonderful knowledge about code blue!”. The detailed debriefing sessions, which followed each simulation, were also appreciated for reinforcing learning and clarifying any doubts. One participant stated, “The interaction and the detailed debriefing”, while another highlighted, “Thorough explanations, detailed steps, valuable debriefs”. This indicates that the post-simulation discussions played a crucial role in consolidating knowledge and providing clarity.

In summary, participants overwhelmingly appreciated the hands-on, realistic nature of the simulations, as it allowed them to practice critical skills in a safe and supportive environment. The interactive format, along with detailed debriefing and clear feedback, was central to the success of the session. Teamwork and collaboration were key elements of the learning experience, with many participants highlighting the value of working together in a group setting. The instructors’ expertise and the well-organized nature of the session contributed significantly to the positive feedback. Overall, the combination of engaging, interactive learning, and practical skill development ensured that the simulation and skills session effectively supported participants in their transition to independent practice.

## 7. Discussion

In this study, the authors evaluated the efficacy of the TIPS program in facilitating the transition of novice nurses to independent and competent practitioners, utilizing the Kirkpatrick Model of Evaluation. Recognizing the importance that transitional programs have on the experience and retention of the new graduate nurses, evaluation of the program is equally as important. The Kirkpatrick model has been an extremely valuable tool to measure the effect of educational initiatives of various scopes and sizes. This is because it utilizes formative and summative evaluation and requires measures of the long-term and system-level impact [27]. The program overall received strong positive reactions, indicating that participants found the content valuable and the delivery methods effective. The high mean score (4.80) indicates that the participants were very satisfied with the program. This suggests that the training materials were engaging and relevant, which is critical for motivating participants to actively engage in learning. The low standard deviation suggests homogeneity in reactions, meaning that the program received consistently high ratings across the groups. This is a strong indication that the training met participants’ needs, was perceived as valuable, and was likely engaging for a broad range of nurses with varying levels of experience and working in different clinical practice settings. Specific topics, such as palliative care (5.0), chest pain management (5.0), and ostomy care (5.0), received perfect scores, reflecting participants’ high satisfaction with these subjects. These areas were likely considered relevant and applicable to their clinical work. Forms and restraints, with a score of 3.8, received the lowest rating, indicating the need for improvement. This could be due to the perceived irrelevance of this topic to some participants or dissatisfaction with the delivery of this session. It suggests a need for review of content or delivery methods for this module. 

Learning gains for Level 2 are clearly evidenced by the increase in average scores across all topics. The difference from pre- to post-test scores reflects a meaningful acquisition of knowledge and skills. Deteriorating Patient/Code Blue simulation, with a change of 1.0, suggests that the simulation was particularly effective in improving nurses’ ability to respond to emergency situations. The Chest Pain Management webinar showed a change of 2.08 points, with a 95% confidence interval (CI) ranging from 2.42 to 4.5, indicating that this topic was highly impactful and that participants gained essential knowledge that is likely to directly influence their clinical decision-making and care delivery. Palliative care, which increased from 3.0 to 4.8, shows a significant learning gain in this critical area, which is essential for providing compassionate care during end-of-life situations. The statistical significance of these changes (e.g., confidence intervals and effect sizes) suggests that these learning outcomes were not due to chance but rather reflect the true impact of the training program. The high effect sizes demonstrate that the program had a strong effect on improving the participants’ clinical knowledge and skills, thereby developing their self-perceived confidence.

According to Bohner’s discussion of Meleis’s Theory, nurses going through a transitional phase in their career may experience a greater impact on their physical and mental wellbeing [10,18]. This can ultimately contribute to a decline in resiliency and pose a threat to wanting to remain in the nursing profession [28]. In this vulnerable state, without the appropriate transition support, nurses are predisposed to enter a state of Transition Shock, which can lead to job dissatisfaction and difficulties adjusting to their professional responsibilities [11,12,13,29]. New graduate nurses are particularly vulnerable when transitioning from academia to practice and have been found to experience a decrease in their self-perceived confidence after starting independent practice [8]. This is concerning, as confidence is strongly associated with the development of communication, ability to handle dynamic situations, professional competence, and overall job satisfaction [30]. Novice nurses rely on performance feedback on their clinical skills and ongoing mentorship to develop their confidence in practice to enhance safe patient care [31]. However, entering the profession while in a state of post-pandemic recovery, where increased patient acuity, staffing shortages, decrease availability of nursing mentors, and capacity pressures in the hospital system are a constant struggle, the development of a new graduate nurse’s confidence may be compromised [8,31]. Several studies examining the impact of the COVID-19 pandemic on the new graduates’ transition to practice have reported higher levels of stress and burnout among this group, related to unmanageable workloads, mismatch of expectations, and a lack of practice readiness [32,33,34].

Having a program like TIPS facilitates transition and supports the overall experience of the new hire and their well-being, ultimately preventing Transition Shock [12]. Transition programs that allow for ample professional development opportunities to practice clinical skills, provide timely feedback, and discuss clinical observations have been found to strongly support the transition of new graduate nurses through its impact on the development of self-perceived confidence [8,17,30]. TIPS comprehensively achieves this through virtual webinars, simulation-based learning and mentorship provided through clinical rounding. Increased confidence scoring from the participants’ post-attendance to webinar and simulation events demonstrates how the TIPS program was able to support the development of confidence for clinical practice. Further, high compliancy scores during all simulation events demonstrated the effect TIPS had on the new nurse’s understanding of their clinical roles and responsibilities, structure and expectations of their clinical environment, and ability to adapt to changing clinical scenarios, all of which have been found to positively impact the professional transition of new graduate nurses [14].

Overall, the data demonstrate the benefit of having a comprehensive and customized training program that blends educational delivery with the availability of clinical mentorship and encompasses the full first year of practice for new graduate nurses. In alignment with the existing literature, nurses who participate in engaging learning opportunities such as webinars, in-services, and simulation and have the availability of clinical mentorship where they can receive timely and applicable feedback on their practice, report a sense of improvement in clinical preparedness, resulting from learning methods that directly relate to patient care delivery and interventions [11,28].

A practice–education gap exists between academia and the healthcare sector. However, programs like TIPS that offer catered transitional support may help bridge this gap. It is important to recognize that further collaboration and communication between academia and the healthcare sector needs to occur for a program like TIPS to have wide-spread and continued success. In a review of a partnership between an academic and healthcare institution, researchers found several benefits, including more rapid transition to practice and improvement in the quality of patient care [35]. The development of a committee or partnership where hospitals collaborate with local colleges and universities to share resources, discuss observed patterns, and provide feedback may help close the practice–education gap, better inform professional development policies, and help programs like TIPS become more catered to the learning needs of the new graduate nurse [35].

## 8. Study Contributions and Limitations

This study makes significant contributions to the literature on nurse transition programs, particularly in the context of supporting new graduate nurses through educational and simulation-based education. The TIPS program, which integrates webinars, simulations, and mentorship, aligns with best practices identified in the existing literature on nurse transition programs, such as enhancing clinical competency, confidence, and retention.

One of the central findings of the study is the significant increase in self-reported confidence levels among participants. This aligns with Meleis’s theory of transition, which highlights the importance of providing adequate support during the transition from academia to practice, preventing the experience of Transition Shock [12,18]. The TIPS program appears to effectively mitigate these feelings by providing targeted interventions that boost self-confidence, thereby supporting nurses’ ability to manage the challenges they face in the early stages of their careers [7,14,15].

The evaluation of the TIPS program was successfully structured around the Kirkpatrick Model, demonstrating its ability to capture comprehensive training outcomes: reaction—participants’ satisfaction, as evidenced by a mean score of 4.80, demonstrated a positive reception to the training sessions [22]; learning—significant gains in confidence were noted, with Cohen’s d = 3.01 indicating a strong effect size, suggesting that the program effectively facilitated knowledge transfer [25]; behavior—post-simulations showed 100% compliance scores, validated by TIPS facilitators and educators, confirming that the learning outcomes were successfully applied in practice; and results—the program’s long-term impact on nurse retention, evidenced by a reduction in turnover rates, fulfilled the highest level of the Kirkpatrick Model, indicating its organizational-level effectiveness. This comprehensive evaluation approach not only highlights TIPS’ success but also offers a rigorous benchmark for other similar programs [3].

The TIPS program offers key strategies that contribute to nurse retention, such as Simulation-Based Education—an approach which emphasized critical thinking and teamwork within realistic clinical scenarios, directly enhancing confidence in clinical practice [36]. Furthermore, by addressing the needs of both novice nurses and experienced mentors, TIPS fostered a feedback loop that strengthened professional development and emotional support through targeted mentorship [11,16]. Finally, the program offered comprehensive transition support, effectively bridging the gap between academic education and clinical practice through interactive webinars and ongoing clinical education, fostering a supportive and inclusive learning environment [30,37]. These strategies collectively promote job satisfaction, competence, and a sense of belonging, critical factors for retaining a stable nursing workforce [36,37,38].

TIPS addresses the unique challenges posed by the post-pandemic nursing environment, including high burnout rates, Transition Shock, and workforce shortages. Many new graduate nurses face significant burnout and Transition Shock, exacerbated by the pandemic’s impact on healthcare systems. The TIPS program’s combination of mentorship and simulation addresses these challenges, promoting emotional well-being and skill development [31]. The program also helps bridge the gap between academic preparation and clinical practice, which has been especially important given elevated patient acuity and workforce shortages [35].

By integrating mentorship, simulations, and collaborative partnerships, TIPS offers a replicable model for improving novice nurse transitions, particularly in resource-constrained environments. The findings underscore the importance of comprehensive, interdisciplinary support systems during career transitions, which have been linked to higher retention and better overall workforce health [11,30].

Overall, the TIPS program exemplifies a robust, evidence-based approach to supporting new nurses, ensuring organizational resilience and contributing to the broader discourse on nurse transition strategies in the post-pandemic era. The results of the TIPS program show promising outcomes in improving confidence and reducing nurse turnover, but several limitations should be acknowledged to provide a more comprehensive understanding of the program’s efficacy and potential areas for improvement.

A key limitation of the study is the absence of a control group, which prevents the researchers from conclusively attributing improvements in confidence and retention solely to the TIPS program. Randomized controlled trials (RCTs) have been identified as the gold standard for evaluating the effectiveness of nurse transition programs, as they control for confounding factors and allow for more definitive conclusions about cause and effect [39]. In light of this, future research should aim to include a control group, as seen in other studies of nurse-education interventions. This approach could strengthen the evidence for the TIPS program by addressing the limitation of selection bias and ensuring that the observed outcomes are due to the intervention itself.

Another limitation is the potential for selection bias in the participant sample. The study indicates that more than half of participants were nursing students, with smaller percentages from other roles, like RNs and RPNs. This could skew the results, as nursing students may have different levels of confidence and expectations compared to more experienced nurses. Selection bias is a recognized issue in transition-program evaluations, as those who choose to participate may already have higher motivation or interest in professional development [18].

In future studies, researchers could minimize selection bias by using random sampling from a larger pool of participants, or by conducting subgroup analyses to explore how different groups (e.g., nursing students versus RNs) benefit from the program. Further, the study’s findings are specific to one healthcare organization, Trillium Health Partners, thus limiting the ability to generalize the results to other settings. Nurse transition programs often face challenges when it comes to scalability, as organizational culture, resources, and patient demographics can all affect the outcomes of such interventions [34].

To enhance generalizability, future studies could replicate the TIPS program in different healthcare settings, including rural hospitals, community clinics, and academic medical centers. By expanding the program to different settings, researchers can better assess universal applicability and identify specific modifications needed for various environments.

The program mentions a decrease in nursing turnover rates within the hospital, which could be influenced by various other factors, such as broader organizational changes or the impact of the COVID-19 pandemic. Uncontrolled external factors are a common challenge in healthcare research, especially when assessing the effectiveness of educational interventions [40,41].

Future research could control such variables by collecting detailed data on other organizational initiatives and using multivariate analysis to isolate the effect of the TIPS program. Additionally, longitudinal studies that track participants over a longer period could help assess the sustained impact of the program on nurse retention, job satisfaction, and performance.

Studies have consistently shown that confidence in clinical skills directly impacts job satisfaction, professional competence, and overall well-being [28,30]. The TIPS program’s success in fostering confidence mirrors findings in the literature, where simulation-based and mentorship-supported transition programs are associated with higher self-perceived competence and better adaptation to clinical roles [40,41]. The study reinforces the growing body of evidence that highlights the crucial role of transition programs in enhancing nurse retention and mitigating the negative effects of role transition.

The study’s findings related to nurse retention are particularly notable. The data demonstrate that nursing turnover rates decreased within 6 months of program participation, with 100% of nursing graduated participants remaining employed within the organization. This finding echoes the results from several studies that emphasize the role of transition programs in improving nurse retention [34]. Nurse turnover, particularly among new graduates, is a significant challenge in healthcare systems globally [42]. By providing a structured, supportive transition experience, the TIPS program appears to directly address the factors that contribute to nurse attrition, such as burnout, stress, and lack of confidence in clinical skills [8,37].

The existing literature also highlights the effectiveness of structured transition programs in reducing turnover. For example, in one study, new graduate nurses who participated in mentorship and educational programs demonstrated higher retention rates and faster adaptation to clinical practice [35]. The current study’s results are consistent with these findings, suggesting that well-designed transition programs can play a critical role in fostering both short-term job satisfaction and long-term retention.

Overall, the study’s contributions are valuable in the context of the existing literature on nurse transition programs, particularly in terms of enhancing confidence, improving retention, and supporting new nurses during the challenging transition from academia to practice. However, its limitations, such as the lack of a control group, selection bias, and limited generalizability, are important to consider for future research. Addressing these limitations, using randomized designs, and expanding the study to multiple settings could provide stronger evidence of the program’s impact and inform future efforts to improve nurse education and retention across diverse healthcare environments.

Furthermore, statistical analysis of the self-reported confidence level of participants for each webinar and simulation were based on averages of participants’ ratings, and hence, may have affected the results. Additionally, the study relied on self-reported data for reaction (Level 1) and learning outcomes (Level 2), which could introduce response bias. Participants may have been inclined to give positive feedback due to social desirability, especially if they perceived that their responses would impact their training evaluations or job performance.

To minimize bias, future studies could incorporate objective measures of learning (e.g., performance evaluations and clinical assessments) and utilize multiple data sources (e.g., preceptor evaluations, patient outcomes, and peer reviews) to provide a more balanced and comprehensive assessment of the program’s effectiveness. Anonymous feedback mechanisms could also help reduce social desirability bias in Level 1 reactions.

Despite the positive contribution of the TIPS program in supporting the retention of skilled nurses at THP and the use of a valid model to evaluate the program, the absence of a control group calls for caution in generalizing the results to other healthcare institutions. Future studies should include a control group to better determine any opportunity to generalize beyond the scope of the TIPS program.

## 9. Opportunities

TIPS has highlighted the possibilities of how this program can continue to grow and further support nurses at THP. The confidence ratings of the participants highlight how this program can be incorporated into the creation of pathways to support nurses upskilling in specialty clinical areas. Participant feedback also suggests that the TIPS program be included in nursing orientation, with some participants strongly recommending that it become a mandatory component of nurse onboarding.

## 10. Conclusions

To effectively support the recruitment and retention of our new and aspiring nurses at THP, a need for more creative and consistent educational offerings and increased clinical support was identified. The TIPS program was developed to address the clinical needs of the nursing workforce. It served as a validated effective strategy to support the safe and seamless transition to independent practice. TIPS has effectively demonstrated that it supports clinical competence and confidence, which are central antecedents to the development of a strong, autonomous professional nursing workforce. Investing in a supportive learning and working environment during this critical transitionary time in their career not only facilitates the development of the above qualities but also fosters a stronger relationship for nurses with the healthcare organization, thereby contributing to improving retention rates. The TIPS program demonstrates how listening to the needs of new and aspiring nurses, in addition to tracking and trending clinical support, has contributed to supporting nursing staff with the knowledge, skill, judgement, and confidence to practice competently.

## Figures and Tables

**Table 1:** Mean scores of confidence level per topic.

TIPS Phase 1: September 2023–March 2024	Mean Scores of Confidence Level	
TIPS Topics	Pre	Post
Chest tube management	2.5	2.5
Deteriorating patient/code blue SIM	3.8	4.8
IV therapy	3.21	4.3
Chest pain management	2.42	4.5
Peripheral, central lines, and chest pain SIM	4.2	5
Enteral therapy	2.9	4.7
Ostomy care	3	5
Foley care	2.8	4.3
CBI care	2.2	4.8
GI/GU SIM	3.5	4.7
Blood derivatives/transfusion	3.07	4.8
Shock	2.28	4.6
Shock SIM	3	4.8
Palliative care	3	4.8
CVADs	2.7	4.4
Palliative care SIM	3	4.9
Delirium and responsive behaviors	2.7	4.5
Restraints and forms	2.5	4.4
Responsive behaviors and code white SIM	3.1	4.1
Total average:	2.9	4.5

## Data Availability

The raw data supporting the conclusions of this article will be made available by the authors upon request.

## Public Involvement Statement

No public involvement in any aspect of this research.

## Guidelines and Standards Statement

This QI project adhered to organizational best practices and ethical guidelines for quality improvement activities, ensuring the protection of participant confidentiality and privacy. All data collected were anonymized, stored securely, and used exclusively for the purpose of performance improvement. The project followed professional ethical standards, ensuring equity, fairness, and confidentiality in line with accepted principles of quality improvement.

## Appendix B. Learning Series Content (Phase One)

**Webinar**	**Simulation**
September 2023	
Oxygen Therapy	None
Chest Tubes	
October 2023	
Intravenous Therapy	None
Chest Pain Management	
November 2023	
Enteral Therapy	Deteriorating Patient and Code Blue
Ostomy Care	Peripheral and Central Lines
December 2023	
Foley Care	GI/GU Simulation
Continuous Bladder Irrigation	
January 2024	
Blood Products/Transfusion	Shock Simulation
Shock Management	
February 2024	
Palliative Care	Palliative Simulation
Central Venous Access Device Care	
March 2024	
Delirium and Responsive Behaviors	Code White Simulation (Responsive Behavior)
Restraints and Forms	

## Appendix C. Learning Series Content (Phase Two)

**Webinar**	**Simulation**
August 2024	
Deteriorating Patient and the Code Blue Process	
1 event	4 events
September 2024	
Cardiac System and the Code Blue Process	
1 event	2 events
October 2024	
Gastrointestinal and Genitourinary Systems and the Code Blue Process	
1 event	3 events
November 2024	
Endocrine System and the Code Blue Process	
1 event	3 events
December 2024	
Mental Health and the Code Blue Process	
1 event	1 event
January 2025	
Neurological System and the Code Blue Process	
1 event	3 events
February 2025	
Respiratory System and the Code Blue Process	
1 event	3 events
March 2025	
Simulation Roulette Random topic from content covered in phase 2 will be selected for nurse to perform, which will support assessment of Kirkpatrick Level 3	
No webinar	4 events

## Appendix F. TIPS Questionnaires

**Pre-Webinar Survey**						
Level 2: Confidence	Questions/Statement	Response				
	I am confident in my ability to perform (webinar topic) in my independent practice.	1. Strongly disagree	2. Disagree	3. Neutral	4. Agree	5. Strongly agree
	I am confident in my ability to seek out support or further education regarding (webinar topic) in my independent practice.					
**Post-Simulation Survey**						
Level 1: Reaction	Questions/Statement	Response				
	I enjoyed the method in which the content was organized.	1. Strongly disagree	2. Disagree	3. Neutral	4. Agree	5. Strongly agree
	The presenter was clear and knowledgeable in their delivery of content.					
Level 2: Confidence	I have gained greater confidence in my ability to perform (webinar topic) in my independent practice.	1. Strongly disagree	2. Disagree	3. Neutral	4. Agree	5. Strongly agree
	I am confident in my ability to seek out support or further education regarding (webinar topic) in my independent practice					
**Open-Ended**						
Post-Webinar	What is one thing that you would like to be done differently in future webinars?					
Post-Simulation	What did you enjoy the most about the webinar and accompanying simulation?					
